# Thermal and Sedimentation Stress Are Unlikely Causes of Brown Spot Syndrome in the Coral Reef Sponge, *Ianthella basta*

**DOI:** 10.1371/journal.pone.0039779

**Published:** 2012-06-22

**Authors:** Heidi M. Luter, Steve Whalan, Nicole S. Webster

**Affiliations:** 1 Australian Institute of Marine Science at James Cook University, James Cook University, Townsville, Queensland, Australia; 2 School of Marine and Tropical Biology, James Cook University, Townsville, Queensland, Australia; 3 Australian Institute of Marine Science, Townsville, Queensland, Australia; 4 Research Institute for the Environment and Livelihoods, Charles Darwin University, Darwin, Northern Territory, Australia; 5 School of Environment, Science and Engineering, Southern Cross University, Lismore, New South Wales, Australia; Utrecht University, Netherlands

## Abstract

**Background:**

Marine diseases are being increasingly linked to anthropogenic factors including global and local stressors. On the Great Barrier Reef, up to 66% of the *Ianthella basta* population was recently found to be afflicted by a syndrome characterized by brown spot lesions and necrotic tissue.

**Methodology/Principal Findings:**

Manipulative experiments were undertaken to ascertain the role of environmental stressors in this syndrome. Specifically, the effects of elevated temperature and sedimentation on sponge health and symbiont stability in *I. basta* were examined. Neither elevated temperature nor increased sedimentation were responsible for the brown spot lesions, but sponges exposed to 32°C developed substantial discoloration and deterioration of their tissues, resulting in death after eight days and a higher microbial diversity in those samples. No shifts in the microbial community of *I. basta* were observed across a latitudinal gradient or with increased sedimentation, with three previously described symbionts dominating the community of all sponges (*Alphaproteobacteria, Gammaproteobacteria* and *Thaumarchaea*).

**Conclusions/Significance:**

Results from this study highlight the stable microbial community of *I. basta* and indicate that thermal and sedimentation stress are not responsible for the brown spot lesions currently affecting this abundant and ecologically important sponge species.

## Introduction

Marine sponges host a diverse range of microorganisms, including 32 bacterial phyla and both major lineages of Archaea [1], [2], [3]. Coupled with their high diversity, sponge microorganisms are involved in important functional processes including nitrification [4], [5], [6], [7], [8], [9], [10], denitrification [7], [11] and Anammox [11], [12]. Given the host specificity of sponge microbes, evidence of vertical transmission of symbionts to larvae (reviewed in [2]) and their involvement in important functional processes, it is clear that sponge-associated bacteria are critical to the health and survival of their host.

The stability of sponge-microbial associations appears to vary between species and environments. For instance, whilst the stable microbial community in *Aplysina aerophoba* is disrupted by disease [13], the symbionts are unaffected by both starvation conditions and antibiotic exposure [14]. Similarly, transplantation of *Aplysina cavernicola* from its original habitat (>40 m) to a shallower, more illuminated environment does not affect the microbial community [15]. In contrast, other species exposed to environmental stress exhibit a disruption of the microbial community including the loss of sponge symbionts and the appearance of putative pathogens. Environmental stressors such as increased temperature [16], [17], [18] and heavy metal exposure [19], [20] have caused shifts in typically stable microbial communities with cascading effects on host health.

Rising temperatures and anthropogenic stressors are being increasingly linked with disease in marine and terrestrial organisms [21], [22]. Disease outbreaks can have extensive impacts on sponge populations and thereby the ecology of reef environments (reviewed in [23]). Notably, an epidemic in 1938 affected 70–95% of the total Caribbean sponge population [24] and disease in commercial Mediterranean species caused the complete collapse of the sponge fishery in the 1980′s [25], [26]. Mass sponge mortalities have also occurred in association with abnormally high seawater temperatures [27], [28], including a recent mass mortality event affecting 80-100% of the *Ircinia fasciculata* populations in the western Mediterranean [29]. The effects of increased sedimentation on sponge communities are not well documented, but there are very clear impacts on host pumping rates [30], [31], [32], metabolism [33], reproductive output [34], [35] and survival of larval recruits [36], [37]. Climate change predicted by the Intergovernmental Panel on Climate Change (IPCC) [38] is likely to have widespread effects on ecosystems such as coral reefs [39], [40] with elevated temperature and terrestrial runoff contributing to disease, as seen in corals exposed to higher temperatures [41], [42], [43] and sedimentation/runoff [21], [44]. While there is compelling evidence for the effects of elevated sea surface temperature and sedimentation on the symbiotic partnerships between coral, zooxanthellae and associated microbes [45], [46], there is a conspicuous absence of knowledge on other benthic groups, such as sponges [47].

A disease-like syndrome is currently affecting a large percentage of *I. basta* in Torres Strait and the Palm Islands in the Great Barrier Reef [48]. However, despite extensive microbial and molecular characterization, no pathogen(s) have been identified or implicated in the formation of brown spot lesions and necrosis in these populations [49] raising questions of whether environmental factors are responsible. This study will explore the role of environmental parameters in the formation of brown spot lesions by assessing the effects of elevated seawater temperature and sedimentation on the sponge holobiont (host and associated microbial communities).

## Materials and Methods

### Sponge collection for analysis of microbial stability

To assess the microbial stability in different populations of *I. basta*, specimens were collected from three different locations in eastern Australia: (1) Orpheus Island, central Great Barrier Reef (18° 36.878' S, 146° 29.990′E), (2) Masig Island, Torres Strait (9° 44.260' S, 143° 25.275′E) and (3) Davies Reef (18° 49.246' S, 147° 37.918′E) representing a geographic range of 1100 km. Seven samples from each location were photographed *in situ* and preserved in 100% ethanol for molecular analysis. All individuals were collected from a 12–15 m depth range.

### Sponge collection for environmental stress experiments

Having determined a uniform microbial community in different *I. basta* populations, fourteen individuals were collected from the fringing reefs of the Palm Islands, central GBR. These donor sponges were cut into smaller explants (approximately 10 cm^3^) using a scalpel blade and transferred to four Aquapurse baskets (TTP plastics by design; Brisbane, Queensland, Australia) that were secured to the reef (18° 35.595′S, 146° 28.955′E). This procedure is well established with sponge explants generally recovering within two months [50]. *I. basta* explants recovered for 12 weeks before collection and transportation to climate-controlled aquaria at the Australian Institute of Marine Science (AIMS), Townsville. *I. basta* explants were placed into 12×30 l flow-through aquaria (flow rate of 600 ml min^−1^) and maintained under a diel cycle of 12∶12 h at 40 µmol quanta m^−2^s^−1^. Seawater was pumped from a pipe 400 m off the coast at the AIMS Cape Cleveland site and filtered to 5 µm to remove large particulates, but still provide sponges with a sufficient food source [51]. Explants were left to acclimate under these aquarium conditions for 48 h before experiments commenced.

### Temperature experiment

To assess the effect of thermal stress on the *I. basta* holobiont, sponge explants were exposed to four different temperature treatments: 27, 30, 31 and 32°C (range +/− 0.2°C). Both 27 and 30°C represent ambient temperatures commonly recorded on inshore reefs of the GBR during the summer period [52] and therefore serve as control temperatures for this experiment. The treatment temperatures of 31 and 32°C represent temperatures which have been linked with mass coral bleaching [52] and the disruption of sponge symbiosis [18].

The experimental design comprised three replicate tanks per temperature treatment, each holding seven explants to allow the destructive sampling of 1 clone per tank for each of the time points. Initially, all tanks were left at 27°C for 72 h and then temperatures were adjusted gradually (0.2°C h^−1^) until reaching the final temperature treatments, to allow sponges to acclimate [18]. Following the acclimation period, explants were randomly selected and removed from each temperature treatment at 0, 1, 4, 7, 14 and 18 days. Day one sampling commenced 24 h after the 32°C treatment temperature had been reached. All sampled explants were photographed to visually assess tissue health and frozen in liquid nitrogen for molecular analysis. After seven days, sponges in the 32°C treatment displayed substantial discoloration and deterioration of the tissues and were considered dead after 8 days. After 14 days, the three remaining temperature treatments were returned to 27°C for the final four days to serve as a recovery period, at which point the remaining explants were sampled.

### Sediment experiment

To examine the effects of increased sedimentation on the *I. basta* holobiont, sediment was collected from the reef slope where *I. basta* was cloned at Orpheus Island (18° 35.595′S, 146° 28.955′E). Sediment was subsequently dried at 100°C and sieved to a final composition of particles that ranged between <63 and ≤180 µm (Table 1). These particle sizes are consistent with suspended sediment compositions for the inner shelf of the GBR [32]. The experimental design comprised four different sediment treatments: 0, 13, 30 and 100 mg l^−1^ (+/−2 mg l^−1^). Following procedures of [53], fiberglass stock tanks (240 l) were used to deliver short, frequent pulses of concentrated sediment stocks to treatment tanks. The stock tanks were designed with a 45° taper at their base to prevent sediment accumulation and ensure constant rates of delivery. To maintain sediment suspension throughout the experiment, an external 2400 l h^−1^ pump (Eheim 1260) was used to circulate the water/sediment suspension from the base to the top of the tank. The delivery of sediment pulses was achieved using a second Eheim 1260 pump, which was set on a timer to pulse for 8 sec every 8 min and delivered approximately 120 ml of concentrated sediment stock to the treatment tanks. A second inverted pump (Eheim Compact+3000 pump) was suspended at the waterline of each experimental tank to further improve re-suspension of sediments. The frequent pulsing and partial re-suspension of sediments ensured that turbidity (NTU) within tanks was typically 10% CV [coefficient of variation, CV  =  (Std Dev x100)/mean] when logged every 30 s and within 5% CV on a daily basis (A. Negri pers. comm.). Total suspended sediment (TSS) concentrations in the treatment tanks were achieved and maintained by measuring turbidity (NTU) with a TPS 90 FL-T water quality logger (Springwood, QLD, Australia). Turbidity measurements were taken daily throughout the experiment and the amount of sediment added to the concentrated stock tanks was adjusted according to the following turbidity calibration equation: NTU = 0.264× TSS +0.05, r2 = 0.99.

**Table 1:** Sediment particle compositions added to concentrated stock tanks and used to dose sponges.

Percentage	Particle size
47	>125 µm <180 µm
43	>63 µm <125 µm
10	<63 µm

Consistent with the temperature experiment, there were three replicate tanks per treatment, each tank holding seven explants. Single explants were randomly selected and removed from each sediment treatment tank at 0, 1, 3, 7 and 9 days. All sampled explants were photographed to visually assess tissue health and frozen in liquid nitrogen for molecular analysis. After seven days the sediment influx was turned off and a 48 h recovery period was established.

### DNA extraction and DGGE

DNA was extracted from all sponge tissue samples from each experiment using the Power Plant DNA Isolation kit, MoBio Laboratories (Carlsbad, CA) according to the manufacturer's protocol. The 16S rRNA gene was amplified by PCR with bacterial primers (1055f: 5'-ATGGCTGTCGTCAGC T-3' and 1392r: 5'-ACGGGCGGTGTGTRC-3') [54]. The reverse primer was modified to contain a 40 bp GC clamp [55]. PCR reactions contained 5 µl dNTP (2.5 mM), 5 µl 10×OptiBuffer, 0.15 µl of each primer (100 pmol µl^−1^), 0.4 µl BSA (10 mg ml^−1^), 3 µl MgCl_2_ (50 mM), 0.5 µl Bio-X-ACT Taq polymerase (Bioline, London, UK) and 1 µl DNA template. Reactions were made up to 50 µl total volume with Milli-Q water. The PCR conditions were: 1 cycle at 95°C for 5 min; 30 cycles at 95°C for 30 sec, 55°C for 1 min, 70°C for 1 min; and a final elongation at 70°C for 10 min. Twenty µl of each sample was added to an 8% w/v polyacrylamide gel containing a 50–70% denaturing gradient of formamide and urea. The gels were run at 60°C for 16 h in 1 x TAE buffer at 75 V using the Ingeny D-code system, stained with 1 x sybr gold for 10 min, visualized under UV illumination and photographed with the Vilber Lourmat ChemiSmart 3000 system. Reference bands from each gel were excised, re-amplified with PCR and checked for correct mobility on a 50–70% gel. PCR products were sequenced by Macrogen Inc. (Seoul, Korea) using the forward primer (1055f).

Due to spatial constraints of the gel, only 46 samples can be visualized at once. Therefore, the 30°C treatment from the temperature experiment and the 13 mg l^−1^ treatment from the sediment experiment were excluded from the DGGE analysis.

### Cloning and sequencing (temperature experiment)

The 16S rRNA gene from three replicate sponges, from the 32°C treatment at day 7, and three replicate control samples at 27°C, was amplified by PCR with universal bacterial primers: 63f 5'-CAGGCCTAACACATG CAA GTC-3' [56] and 1492r 5'- GGT TACCTTGTTACGACT T −3' [57]. PCR reactions contained 10 µl 5x MyTaq Buffer, 0.15 µl of each primer (100 pmol µl^−1^), 0.4 µl BSA (10 mg ml^−1^), 0.25 µl MyTaq DNA polymerase (Bioline, London, UK) and 1 µl DNA. Reactions were made up to 50 µl total volume with Milli-Q water. The PCR conditions were: 1 cycle at 95°C for 1 min; 32 cycles at 95°C for 30 sec, 56°C for 30 sec, 72°C for 2 min; and a final elongation at 72°C for 10 min. PCR products from the control and 32°C treatments were pooled and gel purified following the manufacturers protocol (NucleoSpin Extract II, Scientifix). Purified PCR products were cloned with a TOPO TA cloning kit (Invitrogen, Carlsbad, CA) according to the manufacturer's instructions. Plasmids were checked for inserts by PCR amplification using M13 forward and reverse primers. Restriction digests using *HhaI* and *HaeIII* (New England Biolabs Inc.) were performed to determine operational taxonomic units (OTUs) for each library. Eighty-eight clones from each library were screened and triplicates of each representative OTU from both libraries were sent to Magrogen Inc. (Seoul, Korea) for sequencing using 63f and 1492r as the sequencing primers.

### Phylogenetic analysis

DGGE and clone sequences were compared with available databases using the Basic Local Alignment Search Tool (BLAST) [58] to determine nearest relatives and percent similarity. Clone sequences were submitted to Genbank under the accession numbers JN388026-JN388033. Due to the repetition of DGGE sequences from each of the experiments and the latitudinal comparison, only one representative sequence corresponding to each band was submitted to Genbank (accession numbers JN388012-JN388025). Sequences were checked for chimera formation using Greengenes [59] and all chimeric sequences were removed before tree construction. Sequences were compiled, automatically aligned and manually edited in the ARB software package (http://www.arb-home.de [60]). Initially, trees were calculated with almost complete 16S rRNA (1400 bp) sequences for all close relatives of target sequences using the neighbor-joining and maximum parsimony methods in ARB. Partial sequences were subsequently imported to the tree without changing branch topology using the ARB parsimony-interactive method. The robustness of inferred tree topologies was evaluated after 1000 bootstrap re-samplings of the neighbor-joining data in the PHYLIP program [61]. *Synechoccocus elongatus* was used as an out group for the tree.

### Data analysis

Principal Component Analysis (PCA) using a presence (1)/absence (0) matrix for each of the DGGE bands from both experiments and the spatial analysis comparison was used to analyze microbial communities between treatments. All analyses were carried out using Stastitica 8 (StatSoft 2002).

## Results

### Analysis of geographic variability

**Figure 1:**
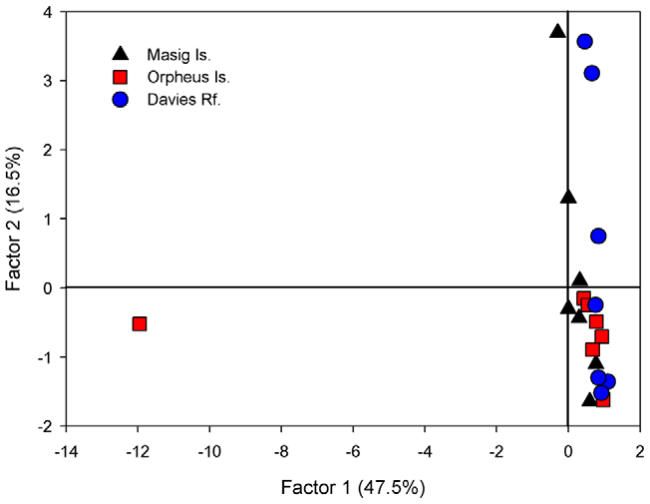
Principal components analysis (PCA) of *I. basta* community composition, using DGGE banding pattern data to construct a similarity matrix of sponges from each of the three geographic locations.

DGGE analysis revealed a microbial community dominated by three microbes, in *I. basta* from each of the three geographical locations (Figure S1). Overall, sponges hosted stable microbial communities regardless of their geographic location. PCA explained 75.4% of the total variation in the first three factors, with the first two factors explaining 64%. The ordination revealed little variation among samples from the three locations, with the exception of one sample from Orpheus Is., which grouped separately (Figure 1). In addition, minor variation was detected between two individuals from Davies Reef and an individual from Masig Is. (Figure 1).

The 16S rRNA gene sequencing of excised DGGE bands from sponges at each of the three locations was the same as previously reported for *I. basta* [49] with the community comprised of *Alpha* and *Gammaproteobacteria* and *Thaumarchaea* (Table 2).

**Table 2:** Sequence similarity in excised 16S rRNA DGGE bands from *I. basta* using BLAST.

Band ID	Accession Number	Percent Similarity	Description
A*	GQ347593	91	α-*proteobacteria* (Oceanic dead zone)
B	FJ205252	89	α-*proteobacteria* (Deep sea hydrothermal region)
C	GQ347593	91	α-*proteobacteria*, (Oceanic dead zone)
D	FJ205252	90	α-*proteobacteria* (Deep sea hydrothermal region)
E	GQ274301	94	γ-*proteobacteria* (marine biofouling sample)
F*	GQ348745	95	γ-*proteobacteria* (oceanic dead zone)
G	FM242455	94	γ-*proteobacteria* (coastal sediment)
H	GQ204920	93	γ-*proteobacteria* (hard coral)
I	JF733387	99	*Pseudomonas* sp. (adult black fly)
J*	EU283427	96	Thaumarchaeota (ascidian)
K	AJ876989	96	Thaumarchaeota (soft coral )
L	EU283427	95	Thaumarchaeota (ascidian)
M	EU182114	99	unidentified bacterium (sediment and seawater, South China Sea)
N	HQ436843	96	unidentified bacterium (Lake Bosten, China)

The asterisks (*) denotes bands from the three dominant symbionts previously reported [47].

### Temperature experiment

**Figure 2:**
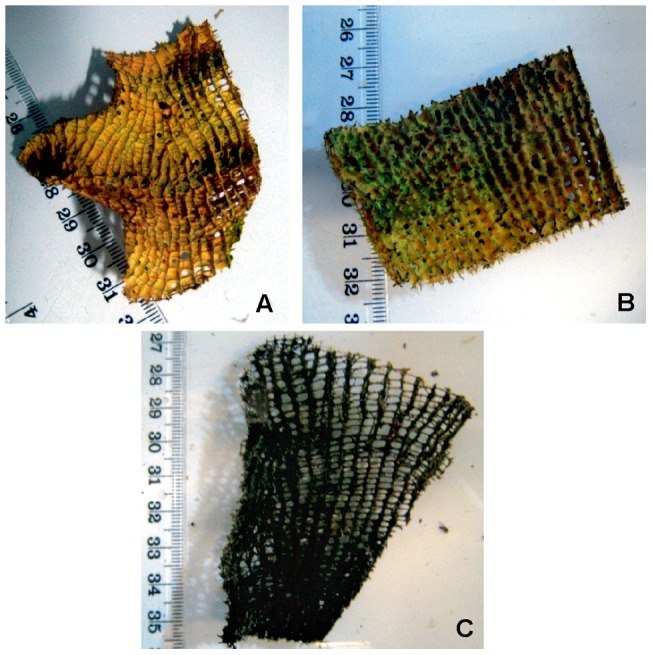
Representative photos of *I. basta* explants from the temperature stress experiment: control explant from 27°C (a), explant from 30°C treatment at day 14 (b) and explant from 31°C treatment at day 14 (c).

*I. basta* explants in all temperature treatments (30, 31 and 32°C) showed consistent color and gross morphologies to those maintained at 27°C for the first four days of the experiment, with no evidence of the brown spot lesions previously described [48], [49]. Explants from the 30°C treatment survived the full 14 day exposure and subsequent four day recovery period, never changing in appearance from the explants maintained at 27°C (Figure 2A, B). Up until day seven, explants from the 31°C treatment also appeared visually similar to control sponges; however, by day 14 they displayed a blackish discoloration, and tissue had retracted resulting in obvious subdermal gaps between skeletal fibers (Figure 2C). Although temperature treatments were returned to 27°C for a 4 day recovery period, blackened explants from the 31°C treatment did not return to their original yellow color and subdermal gaps were still evident.

**Figure 3:**
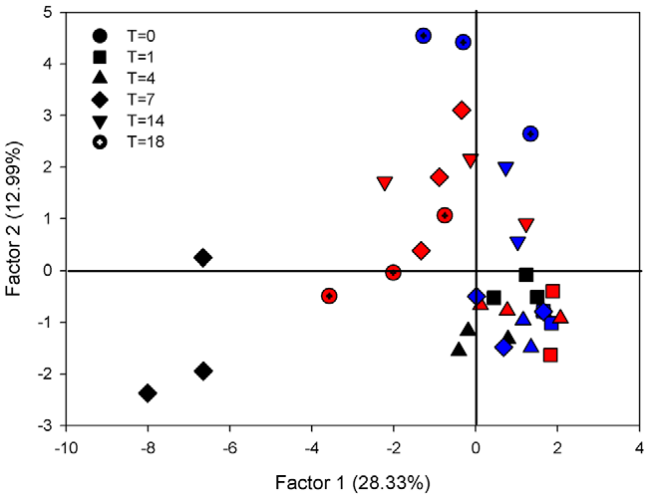
Principal components analysis (PCA) of *I. basta* community composition, using DGGE banding pattern data to construct a similarity matrix, at 27 (blue), 31 (red) and 32°C (black). Symbols correspond to the different time points. Due to spatial constraints of the gel, the 30°C treatment was excluded from the analysis.

Consistent with the sponges sampled as part of the geographic survey, DGGE analysis of *I. basta* from the temperature experiment revealed three dominant bright bands, with additional minor light bands also present (Figure S2). PCA analysis explained 50.5% of the total variation in the first three factors, with the first two factors explaining 41.3%. The ordination revealed a stable microbial community in sponges from all replicate time points and treatments up to and including 31°C. In contrast, all three replicate sponges exposed to 32°C clustered separately after 7 days (Figure 3). Overall, the community composition of *I. basta* explants remained stable over all temperatures for the first four days of the experiment (Figure 3). With the exception of *I. basta* explants at 27°C at day seven, there was larger variation between the three individual replicates for each of the temperature treatments after day four (Figure 3).

The 16S rRNA sequencing of excised DGGE bands from the temperature experiment confirmed the same community previously reported for *I. basta* [49], with the three dominant symbionts present in all treatments (*Alphaproteobacteria*, *Gammaproteobacteria*, and *Thaumarchaea*).

**Figure 4:**
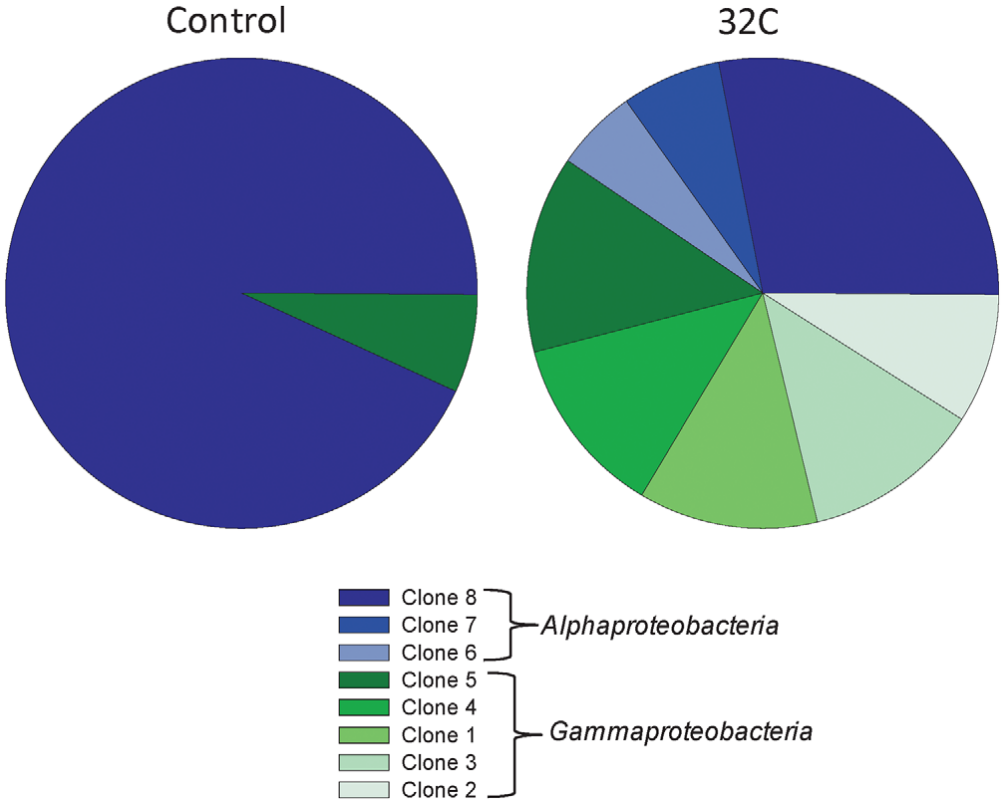
Pie charts showing the differences in bacterial community composition in *I. basta* between control samples and samples from the 32°C treatment at day seven. The graphs were constructed based on the frequency of clones from each library (n = 88 per library). Clone 8 and 5 had identical 16S rRNA sequences in both libraries.

Clone library analysis of *I. basta* from the 32°C treatment at day seven and control samples confirmed the presence of only a few dominant symbionts indicated by DGGE. All clone sequences fell into only two classes, the *Alpha*- and *Gammaproteobacteria*, with both libraries dominated by a single *Alphaproteobacteria* sequence (clone 8). This *Alphaproteobacteria* accounted for 93% of the control library and 28% of the 32°C library. The remaining 7% of the control library was comprised of a single *Gammaproteobacteria* sequence (clone 5), which made up 14% of the 32°C library. The 32°C library contained an additional two *Alphaproteobacteria* sequences (Clones 6 & 7) and four *Gammaproteobacteria* sequences (Clones 1 to 4). *Alphaproteobacteria* and *Gammaproteobacteria* comprised 40 and 60% of the 32°C library respectively (Figure 4).

**Figure 5:**
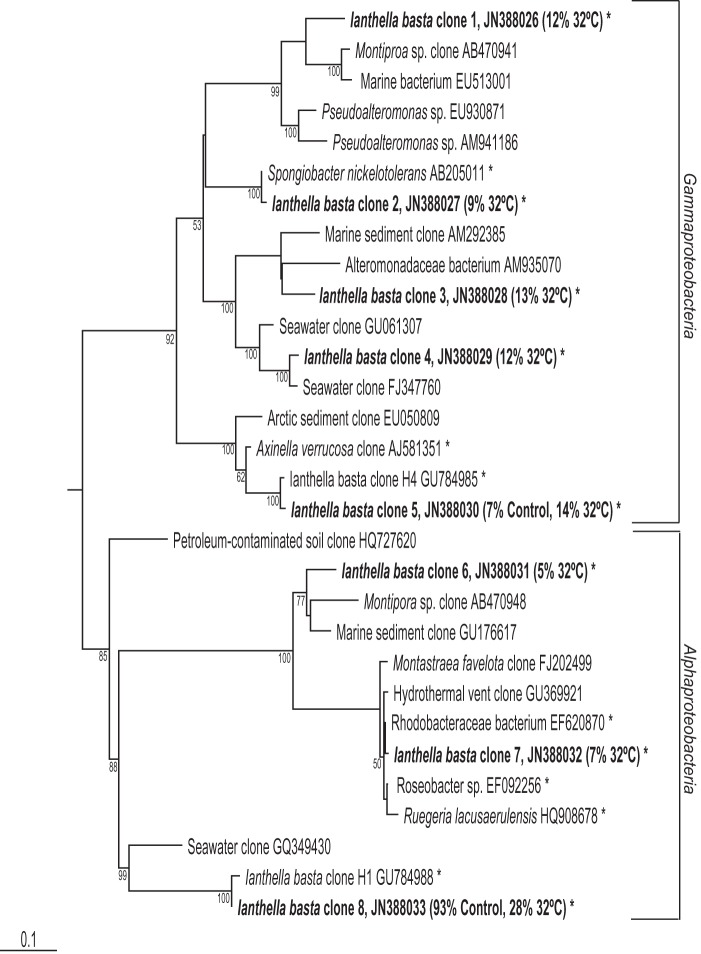
Maximum-likelihood phylogenetic tree from analysis of all 16S rRNA gene sequences retrieved from clone library analysis. *I. basta* sequences from this study indicated by bold font, with the percentage of each library it comprised listed in parenthesis afterwards and * indicates clones isolated from marine sponges. The numbers at the nodes are percentages indicating the levels of bootstrap support based on analysis of 1000 re-sampled data sets. Only values >50% are shown. Scale bar represents 0.1 substitutions per nucleotide position.

Phylogenetic analysis revealed that clone 8 was 99% similar to the previously described *Alphaproteobacteria* symbiont from healthy and diseased *I. basta* (GU784988) [48]. The next closest relatives of clone 8 were only 86% similar, comprising sequences from seawater and sediment samples, as well as other sponge and coral-derived sequences (Figure 5). Clone 5 was identical to the previously described *Gammaproteobacteria* symbiont from *I. basta* (GU784985). Clones 6 and 7, exclusively detected in the 32°C library, were both most closely related to other sponge and coral-derived sequences (Figure 5). The additional *Gammaproteobacteria* sequences observed in the 32°C library were most closely related to other environmental sequences. Specifically, clone 1 was related to a sequence retrieved from the coral *Montipora* sp. (AB470941) while clone 2 was most closely related to *Spongiobacter nickelotolerans* (AB205011) isolated from a marine sponge. Clones 3 and 4 were most closely related to soil and seawater bacteria respectively (Figure 5).

### Sediment experiment

*I. basta* explants in all sediment treatments (13 to 100 mg l^−1^) survived the seven day exposure and further two day recovery period and appeared visually similar to those explants maintained at 0 mg l^−1^. Explants from the 30 and 100 mg l^−1^ treatment accumulated sediments on their surfaces; however, tissue health remained similar to explants maintained at 0 mg l^−1^ when sediments were manually removed after 7 days. None of the sponge clones developed brown spot lesions during the course of the sedimentation experiment.

**Figure 6:**
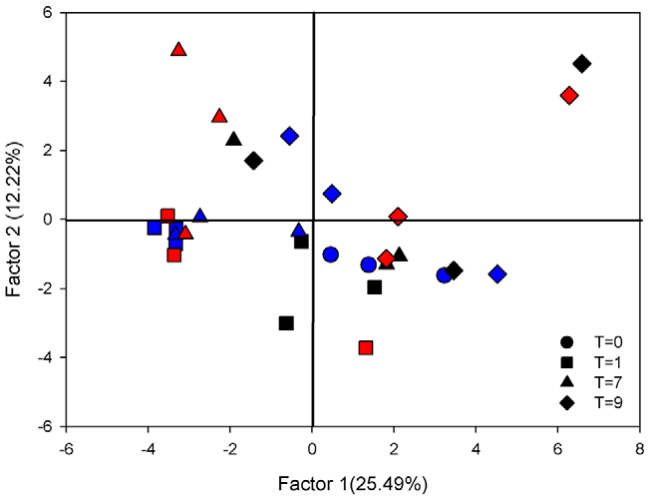
Principal components analysis (PCA) of *I. basta* community composition, using DGGE banding pattern data to construct a similarity matrix, at 0 (blue), 30 (red) and 100 mg l^−1^ (black). Symbols correspond to the different time points. Due to spatial constraints of the gel the 13 mg l^−1^ treatment was excluded from the analysis.

DGGE analysis of sponges in the sediment treatments at each time point confirmed the presence of three dominant community players with other low abundance microbes also present. PCA analysis explained 47.8% of the total variation in the first three factors, with the first two factors explaining 37.7%. In general, there was a large amount of variation between individuals, regardless of the sediment treatment or time point (Figure 6). This variation was attributed to the absence of bands in some samples (see Figure S3) rather than the appearance of novel microbes not normally detected in *I. basta*. 16S rRNA sequencing of excised DGGE bands revealed the same microbes detected in sponges from the temperature experiment with all sequences identical to those previously reported for *I. basta* [49].

## Discussion

Elevated seawater temperature and sedimentation do not appear to be responsible for the syndrome of brown spot lesions currently affecting a large portion of the *I. basta* population on the Great Barrier Reef [48]. Sponges exposed to elevated temperature and sedimentation did not develop symptoms of the syndrome and the microbial symbionts were not affected by the environmental stressors. However, *I. basta* do not tolerate seawater temperatures of 32°C, which is only 2–4 degrees higher than the average summer temperature at Orpheus Is. [52]. This indicates a very narrow thermal threshold for *I. basta* as no morphological changes were evident at 30°C, but sponges at 32°C exhibited substantial discolouration and deterioration of the tissue with death occurring after 8 days. In addition, sponges exposed to 31°C showed symptoms of tissue regression and discolouration after 14 days.

Accompanied with the decline in host health at 32°C, sponges from this treatment had a greater diversity of microbes within the *Alpha*- and *Gammaproteobacteria*. Whilst other studies have reported microbial shifts in sponges exposed to elevated seawater temperatures [16], [18], [62], these studies have described both the loss of symbionts and the appearance of foreign microbes including putative pathogens. Bacteria within the *Bacteroidetes, Epsilon-* and *Deltaproteobacteria* commonly occur after stress in both sponges and corals [18], [63], [64]. In contrast, all three dominant *I. basta* symbionts were maintained in sponges at 32°C after 7 days and all of the microbes exclusively detected in the 32°C treated sponges were members of the *Alpha* and *Gammaproteobacteria*. Given this apparent stability in the microbial community of *I. basta*, it is likely these microbes play an important functional role(s) in this sponge.

Sponges can produce secondary metabolites to maintain their inherent microbial community, as well as protecting themselves from microbial attack [65]. *I. basta* produces both araplysillin and bastadin compounds that are known to have strong biological activity and antimicrobial properties [66], [67]. DGGE analysis revealed higher diversity in moribund samples from 32°C at day seven than from 32°C at day four. It is possible that this increased diversity coincides with the breakdown in *I. basta'*s antimicrobial compound production, allowing the proliferation of a greater number of opportunistic bacteria prior to sponge mortality.

*I. basta* explants exposed to high levels of sediments (100 mg l^−1^) appeared visually similar to those maintained at 0 mg l^−1^, indicating this sponge is capable of withstanding high levels of sediment exposure, at least in the short term. Despite the potential pumping implications from the high amounts of sediment on the sponge surface [30], [31], [33], *I. basta* showed no visible adverse effects. Conversely, coral tissues exposed to high sedimentation rates show decreased photosynthetic activity and necrosis [68]. The ability of *I. basta* to cope with short term sediment stress is likely linked to their habitat and distribution, which includes inshore silty patches and fringing reef slopes [48], [69]. For instance, the mean max turbidity of Orpheus Island in the summer is 26 +/−11 NTU (AIMS Water Quality Monitoring Data), which is equivalent to the turbidity experienced by *I. basta* in the highest treatment of this study. No microbial community shifts were detected in sponges from any of the sediment treatments or time points. While previous studies have documented the negative impacts of water quality and increased sedimentation on sponge pumping rates [30], [31], [32], reproduction [34], [35] and metabolism [33], this is the first study to assess the effects of increased sedimentation on sponge symbiosis.

Sponge microbial communities are generally stable across temporal and spatial scales [14], [70], [71], [72]. For instance, the sponge *Cymbastela concentrica* maintains a similar microbial community composition across a 500 km region of its temperate range [71]. The microbial community of *I. basta* is no exception, with consistent communities observed in sponges from three separate locations across an 1100 km latitudinal gradient.

*I. basta's* microbial community is consistently dominated by three symbionts, regardless of its disease status [49], the environmental stress or the geographic location it inhabits. In all instances, the community is dominated by three symbionts within the *Alphaproteobacteria, Gammaproteobacteria* and *Thaumarchaea* although other low abundance microbes within the *Cyanobacteria, Chloroflexi* and *Verrucomicrobia* are also present [65], [73]. To date, none of the three dominant symbionts have been successfully cultivated [49]. Neither increased temperature nor sedimentation induced the disease-like symptoms (brown spot lesions) observed in *I. basta* in the field [48], indicating that these two environmental factors are unlikely causes of the syndrome. In addition, multiple techniques (e.g. bacterial cultivation, molecular community analysis and electron microscopy) have failed to identify any microbial community shifts or putative pathogens responsible for this syndrome [49]. Given these results, the cause of the syndrome is still uncertain. It cannot be ruled out that other environmental factors (e.g. elevated nutrients) may be responsible, or that it is a combination of different environmental factors working together to induce the lesions. In addition, the pre-filtration of the experimental water (5 μm) may have removed potential protist vectors and/or disease causing protists. Future work will assess the role of protists in disease aetiology and examine immune dysfunction and senescence in the sponge host by identifying differentially expressed genes in sponges displaying the syndrome. This may provide further insight into the cause of the brown spot lesions in this abundant and ecologically important species.

## Supporting Information

Figure S1 **DGGE gel image of 16S rRNA-defined bacterial populations from** ***I. basta*** **from three geographical locations.** Bands excised for sequencing are labeled on the right hand side of the bands, and asterisks (*) denote bands that yielded low sequence quality.
(PPT)

Figure S2 **DGGE gel image of 16S rRNA-defined bacterial populations from** ***I. basta*** **explants in the 27, 31 and 32°C treatments over the course of the experiment (T = 0, 1, 4, 7, 14 and 18).** Bands excised for sequencing are labeled on the right hand side of the bands, and asterisks (*) denote bands that yielded low sequence quality. Due to spatial constraints of the gel, only 46 samples can be visualized at once. Therefore, the 30°C treatment was excluded from the analysis.
(PPT)

Figure S3 **DGGE gel image of 16S rRNA-defined bacterial populations from** ***I. basta*** **explants in the 0, 30 and 100 mg l^−1^ treatments over the course of the experiment (T = 0, 1, 4 and 7).** Bands excised for sequencing are labeled on the right hand side of the bands, and asterisks (*) denote bands that yielded low sequence quality. Due to spatial constraints of the gel, only 46 samples can be visualized at once. Therefore, the 13 mg l^−1^ treatment was excluded from the analysis.
(PPT)
