# Supplementary figures and images for: Thermal and Sedimentation Stress Are Unlikely Causes of Brown Spot Syndrome in the Coral Reef Sponge, Ianthella basta
Source: PLoS One. 2012 Jun 22;7(6):e39779. doi: 10.1371/journal.pone.0039779 (PMC3382149; doi:10.1371/journal.pone.0039779)

## Slide 1
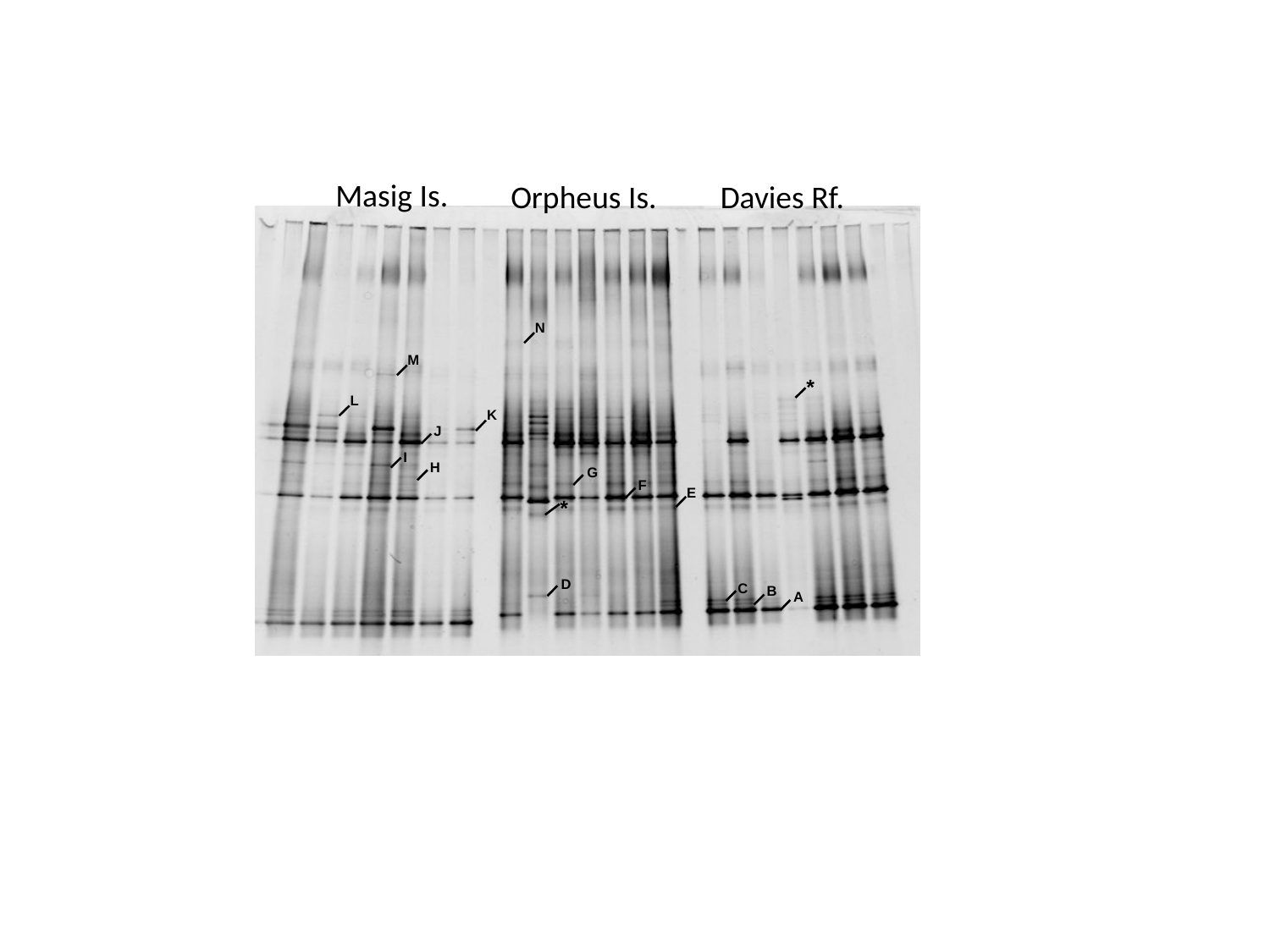

Masig Is.
Orpheus Is.
Davies Rf.
N
M
*
L
K
J
I
H
G
F
E
*
D
C
B
A

## Slide 2
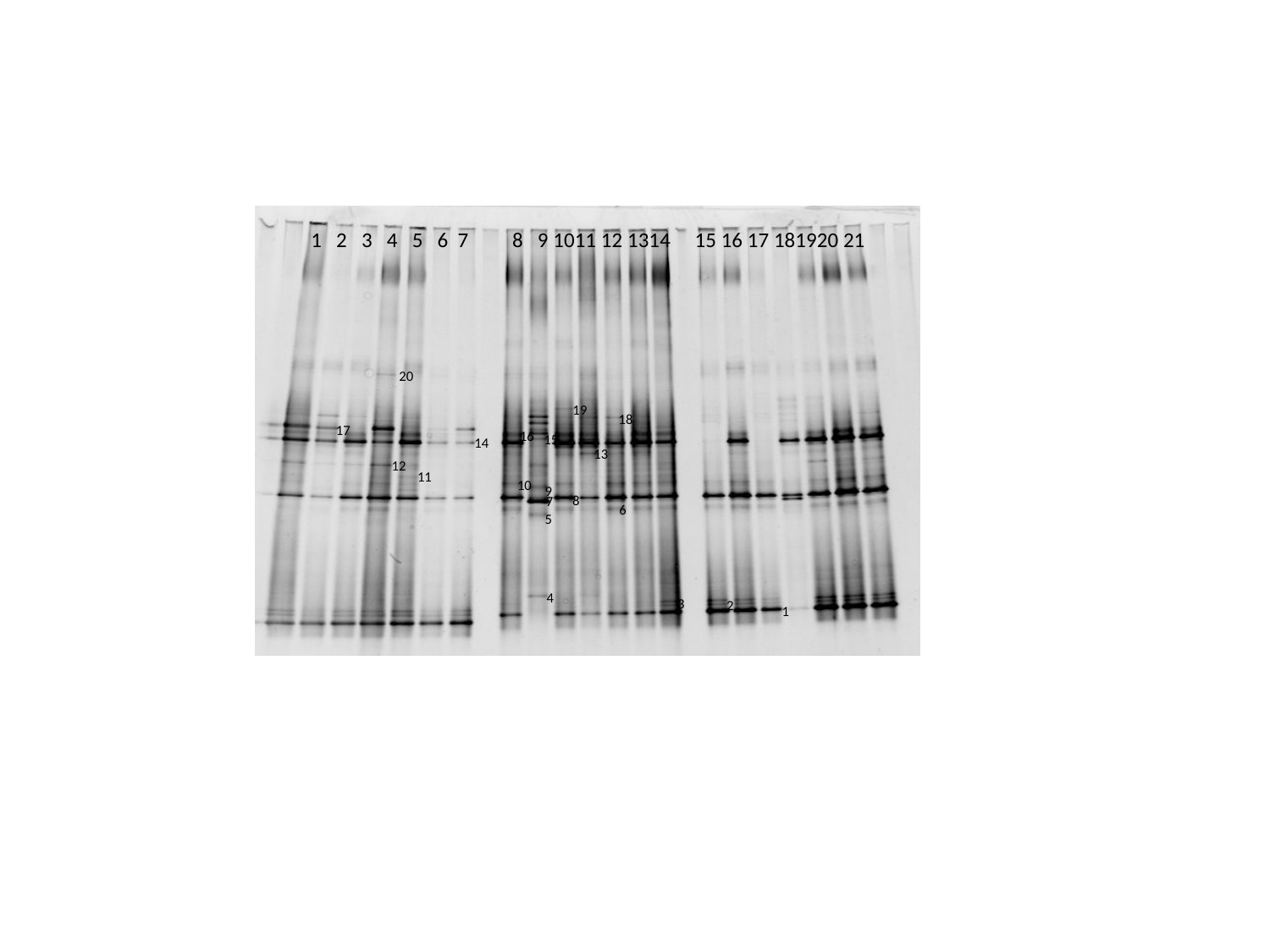

1 2 3 4 5 6 7 8 9 1011 12 1314 15 16 17 181920 21
20
19
18
17
16
15
14
13
12
11
10
9
8
7
6
5
4
3
2
1

## Slide 3
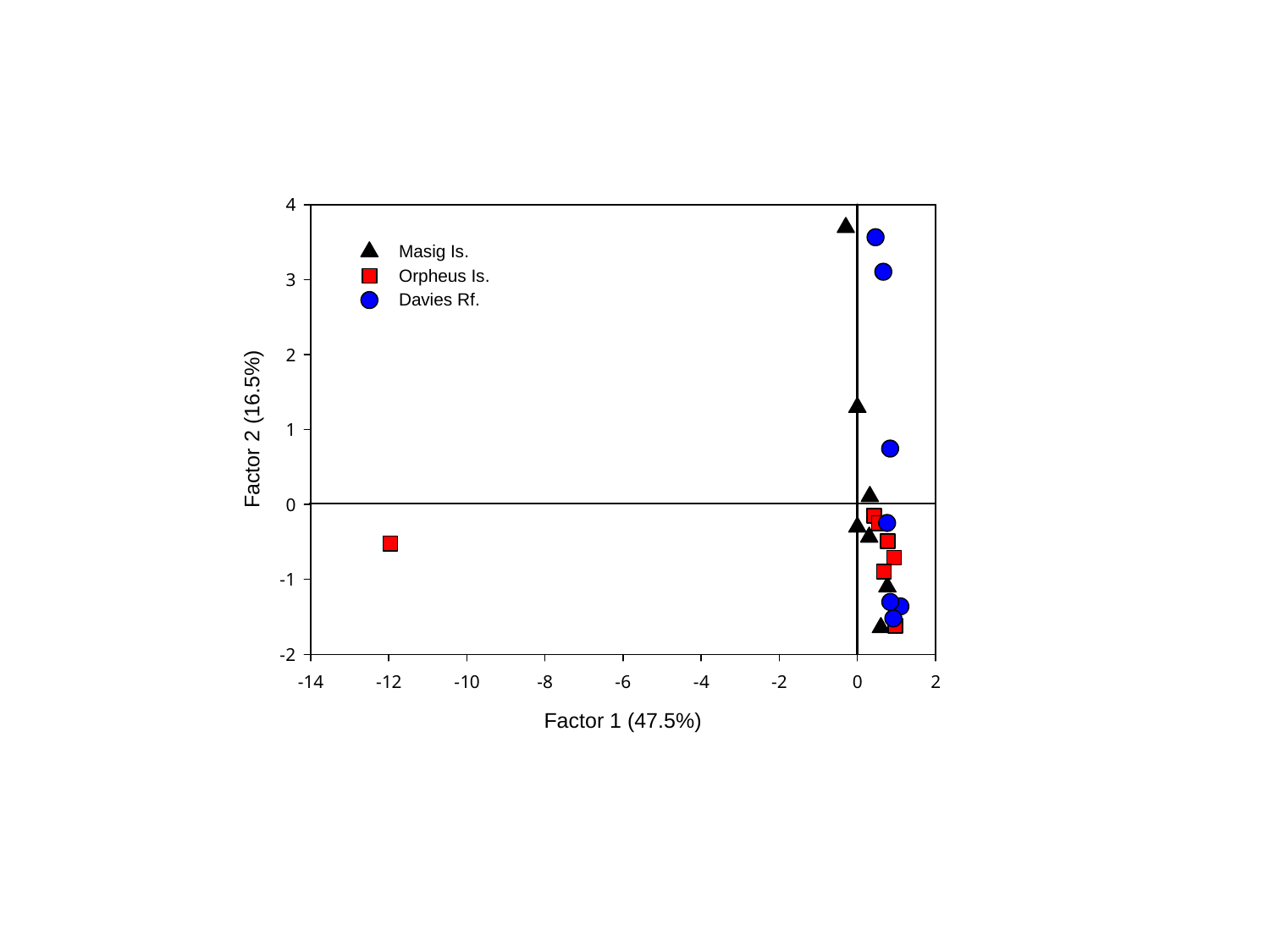

Supplement: Figure S1 — DGGE gel image of 16S rRNA-defined bacterial populations from I. basta from three geographical locations. Bands excised for sequencing are labeled on the right hand side of the bands, and asterisks (*) denote bands that yielded low sequence quality. (PPT) [file pone.0039779.s001.ppt]

## Slide 1
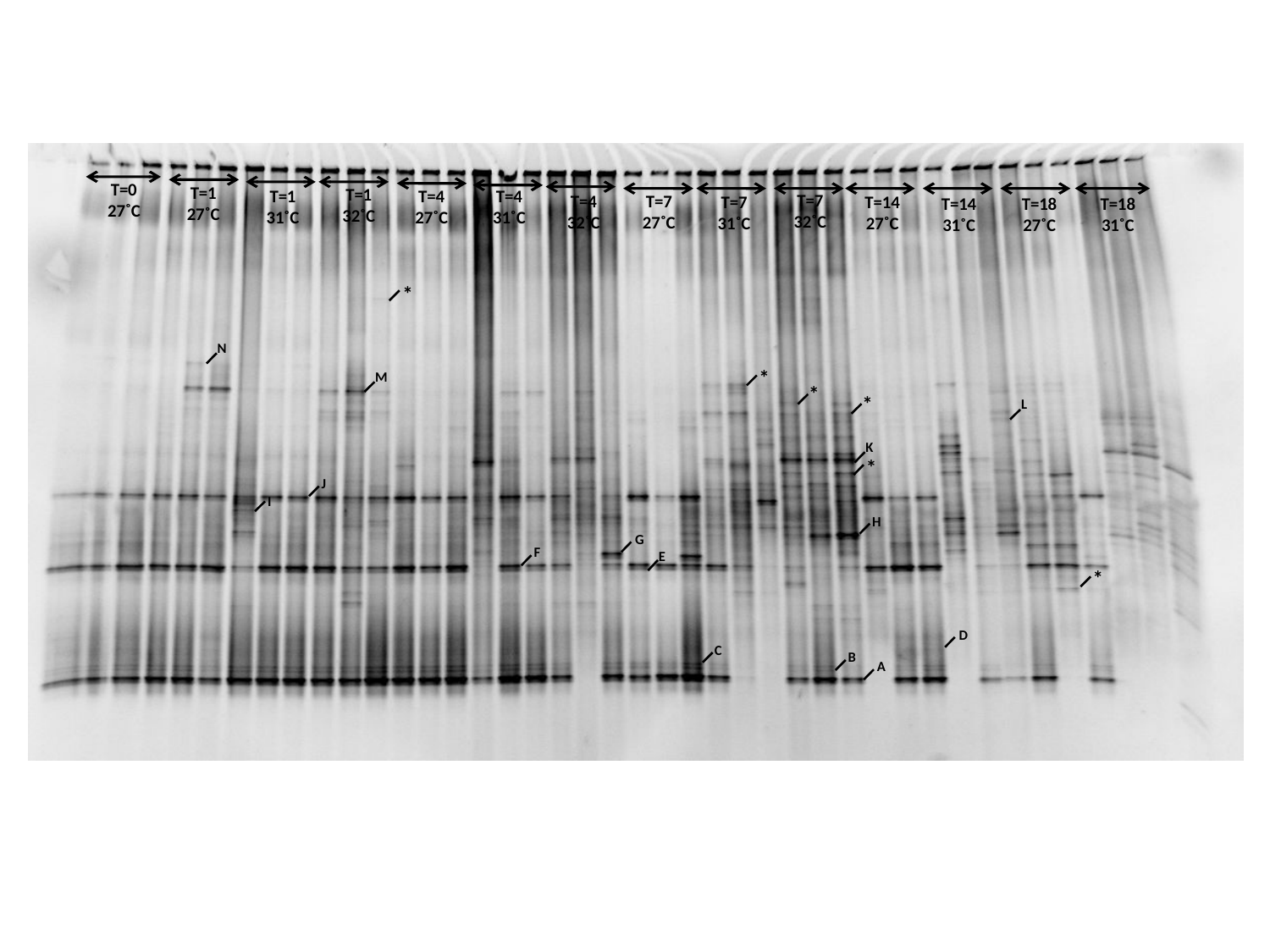

T=0 27˚C
T=1 27˚C
T=1 32˚C
T=4 31˚C
T=1 31˚C
T=4 27˚C
T=7 32˚C
T=4 32˚C
T=7 27˚C
T=7 31˚C
T=14 27˚C
T=18 27˚C
T=14 31˚C
T=18 31˚C
N
*
M
*
*
L
K
*
J
I
H
G
F
E
C
B
A
*
*
D

Supplement: Figure S2 — DGGE gel image of 16S rRNA-defined bacterial populations from I. basta explants in the 27, 31 and 32°C treatments over the course of the experiment (T = 0, 1, 4, 7, 14 and 18). Bands excised for sequencing are labeled on the right hand side of the bands, and asterisks (*) denote bands that yielded low sequence quality. Due to spatial constraints of the gel, only 46 samples can be visualized at once. Therefore, the 30°C treatment was excluded from the analysis. (PPT) [file pone.0039779.s002.ppt]

## Slide 1
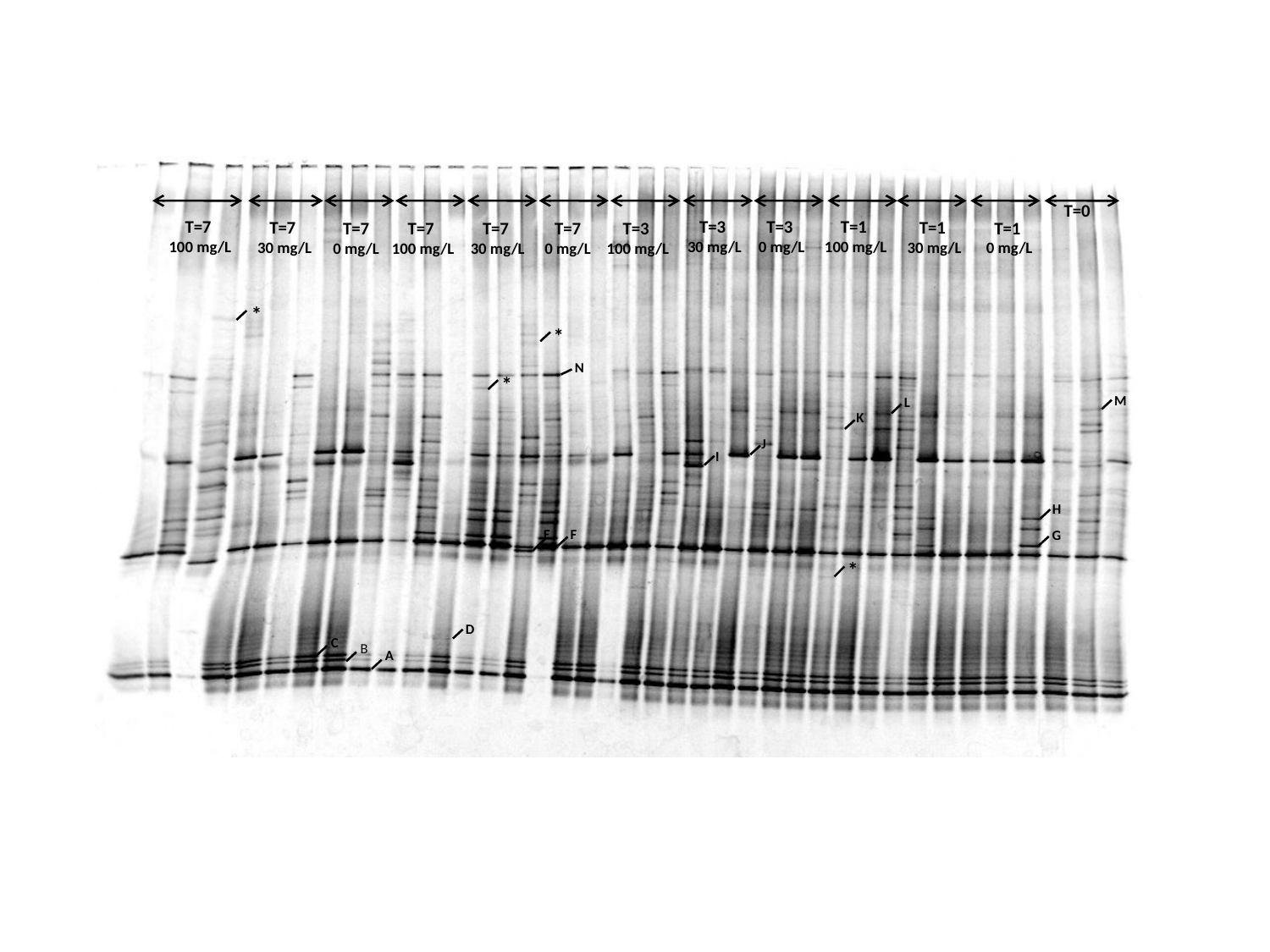

T=0
T=7
100 mg/L
T=3
30 mg/L
T=3
0 mg/L
T=1
100 mg/L
T=1
30 mg/L
T=7
30 mg/L
T=1
0 mg/L
T=7
0 mg/L
T=7
100 mg/L
T=7
30 mg/L
T=7
0 mg/L
T=3
100 mg/L
N
*
M
L
K
J
I
H
F
E
G
C
B
A
*
*
*
D

Supplement: Figure S3 — DGGE gel image of 16S rRNA-defined bacterial populations from I. basta explants in the 0, 30 and 100 mg l−1 treatments over the course of the experiment (T = 0, 1, 4 and 7). Bands excised for sequencing are labeled on the right hand side of the bands, and asterisks (*) denote bands that yielded low sequence quality. Due to spatial constraints of the gel, only 46 samples can be visualized at once. Therefore, the 13 mg l−1 treatment was excluded from the analysis. (PPT) [file pone.0039779.s003.ppt]
